# Factors associated with sexually transmitted infections in sugarcane cutters: subsidies to caring for*

**DOI:** 10.1590/1518-8345.3425.3306

**Published:** 2020-06-19

**Authors:** Juliana Pontes Soares, Sheila Araújo Teles, Karlla Antonieta Amorim Caetano, Thaynara Ferreira Amorim, Maria Eliane Moreira Freire, Jordana de Almeida Nogueira, Brunna Rodrigues de Oliveira, Oriana Deyze Correia Paiva Leadebal, Patricia da Silva Araújo, Ana Cristina de Oliveira e Silva

**Affiliations:** 1 Universidade Federal da Paraíba, João Pessoa, PB, Brazil.; 2 Universidade Federal de Goiás, Faculdade de Enfermagem, Goiânia, GO, Brazil.; 3 Secretaria do Estado de Goiás, Hospital de Doenças Tropicais, Goiânia, GO, Brazil.; 4 Universidade Federal da Paraíba, Departamento de Enfermagem Clínica, João Pessoa, PB, Brazil.; 5 Universidade Federal de Goiás, Instituto de Patologia Tropical em Saúde Pública, Goiânia, GO, Brazil.; 6 Hospital Universitário Lauro Wanderley, Clinica de Doenças Infecciosas e Parasitárias, João Pessoa, PB, Brazil

**Keywords:** Sexually Transmitted Diseases, Prevalence, Vulnerable Populations, Rural Population, Nursing Care, Risk Factors, Doenças Sexualmente Transmissíveis, Prevalência, Populações Vulneráveis, População Rural, Cuidados de Enfermagem, Fatores de Risco, Enfermedades de Transmisión Sexual, Prevalencia, Poblaciones Vulnerables, Población Rural, Atención de Enfermería, Factores de Riesgo

## Abstract

**Objective:**

to estimate the prevalence of Sexually Transmitted Infections (STIs) and associated factors in sugarcane cutters.

**Method:**

a cross-sectional, analytical study with 937 sugarcane cutters from Paraíba and Goiás, states of Brazil, respectively. An outcome variable was the positive results in some rapid tests for HIV, syphilis, hepatitis B and C. Bivariate and multiple analyses were performed to identify the association between these infections and sociodemographic and behavioral variables.

**Results:**

all participants were male, most were young adults and had low schooling. Prevalence of STI was estimated at 4.1% (95% CI: 3.0-5.5). According to multiple regression analysis, the variables age over 40 years (OR 5.0; CI 95%: 1.8-14), alcohol consumption (OR 3.9; CI 95%: 1.3-11.9), and illicit drugs (OR 2.9; CI 95%: 1.3-6.3) were factors associated with the STIs investigated. On the other hand, having some religion (OR 0.4; CI 95%: 0.2-0.8), and work in the Midwest Region (OR 0.4; CI 95%: 0.2-0.9) were factors negatively associated with these infections.

**Conclusion:**

presence of risk behaviors for STI among sugarcane cutters. Screening for these infections in groups of rural workers is essential for early diagnosis and breaking the chain of transmission.

## Abstract (pt)

**Objetivo:**

estimar a prevalência de Infecções Sexualmente Transmissíveis (IST) e fatores associados em cortadores de cana-de-açúcar.

**Método:**

estudo transversal, analítico, com 937 cortadores de cana-de-açúcar dos estados da Paraíba e de Goiás, no Brasil. Considerou-se como variável de desfecho a positividade em algum teste rápido para HIV, sífilis, hepatite B e C. Análises bivariada e múltipla foram realizadas para identificar associação entre essas infecções e variáveis sociodemográficas e comportamentais.

**Resultados:**

todos os participantes eram do sexo masculino, a maioria eram adultos jovens e possuíam baixa escolaridade. Estimou-se uma prevalência para IST de 4,1%(IC 95%: 3,0-5,5). De acordo com a análise de regressão múltipla, variáveis como idade acima de 40 anos (OR 5,0; IC 95%: 1,8-14), consumo de álcool (OR 3,9; IC 95%: 1,3-11,9) e de drogas ilícitas (OR 2,9; IC 95%: 1,3-6,3) foram fatores associados às IST investigadas. Por outro lado, ter alguma religião (OR 0,4; IC 95%: 0,2-0,8) e trabalhar na Região Centro-Oeste (OR 0,4; IC 95%: 0,2-0,9) foram fatores associados negativamente a essas infecções.

**Conclusão:**

presença de comportamentos de risco para as IST entre os cortadores de cana-de-açúcar. O rastreio destas infecções em grupos de trabalhadores rurais é fundamental para diagnóstico precoce e quebra da cadeia de transmissão.

## Abstract (es)

**Objetivo:**

estimar la prevalencia de infecciones de transmisión sexual (ITS) y factores asociados en cortadores de caña de azúcar.

**Método:**

estudio analítico transversal con 937 cortadores de caña de azúcar de los estados de Paraíba y Goiás, en Brasil. Se consideró como variable el resultado positivo en algunas pruebas rápidas para VIH, sífilis, hepatitis B y C. Se realizaron análisis bivariados y múltiples para identificar una asociación entre estas infecciones y las variables sociodemográficas y de comportamiento.

**Resultados:**

todos los participantes eran hombres, la mayoría eran adultos jóvenes con bajo índice de escolaridad. Se estimó una prevalencia de ITS de 4.1% (IC 95%: 3.0-5.5). Según el análisis de regresión múltiple, variables como edad superior a 40 años (OR 5.0; IC 95%: 1.8-14), consumo de alcohol (OR 3.9; IC 95%: 1.3- 11.9) y drogas ilícitas (OR 2.9; IC 95%: 1.3-6.3) fueron factores asociados a las ITS investigadas. Por otro lado, factores como tener alguna religión (OR 0.4; IC 95%: 0.2-0.8) y trabajar en la región Centro-Oeste (OR 0.4; IC 95%: 0.2-0.9) tuvieron una asociación negativa en relación a estas infecciones.

**Conclusión:**

se advirtió la presencia de comportamientos de riesgo para ITS entre cortadores de caña de azúcar. La detección de estas infecciones en grupos de trabajadores rurales es esencial para la detección temprana de diagnóstico y para romper la cadena de transmisión.

## Introduction

Worldwide, approximately one million people acquire some sexually transmitted infection (STI) each day, with an annual forecast of 357 million new curable infections. There are more than 20 microorganisms causing STIs, among those responsible for asymptomatic infections are hepatitis B virus (HBV), hepatitis C virus (HCV), human immunodeficiency virus (HIV) and the *Treponema pallidum*, the aetiological agent of syphilis^(^ 1 ^)^.

The World Health Organization (WHO) has estimated 257 million and 71 million people chronically infected with HBV and HCV in 2015, respectively. These viruses are responsible for more than one million deaths per year and are the cause of acute and chronic liver disease, including cirrhosis and hepatocellular carcinoma^(^ 2 ^)^. Brazil is considered a country of low endemicity for viral hepatitis B and C, however, considering its population size, the absolute number of infected individuals is significant. According to the epidemiological bulletin for 2019, 233,027 cases of hepatitis B and 359,673 cases of hepatitis C were reported from 1999 to 2018^(^ 3 ^)^.

Globally, about 37.9 million people were living with HIV in 2018, with 1.7 million new infections^(^ 4 ^)^. In Brazil, the HIV epidemic is of the concentrated type, that is, it mainly affects key populations. Most cases are detected in urban regions, although a gradual process of an epidemic’s growth in the countryside has been observed in the country. From 1980 to June 2019, 966,058 cases of AIDS were notified in the country. In 2018, 43,941 new cases of HIV infection were registered^(^ 5 ^)^.

In 2016, the number of syphilis cases in people aged 15 to 49 was estimated at 6.3 million, with a global prevalence of 0.5%^(^ 6 ^)^. In Brazil, in recent years, there has been an increase in the number of people infected by *T. pallidum*. In 2017, the detection rate of acquired syphilis was 59.1 cases/100,000 inhabitants, while in 2018 this rate reached 75.8 cases/100,000 inhabitants, an increase of almost 17 new cases/100,000 inhabitants in just one year^(^ 7 ^)^. HIV, HBC, HCV, and *Treponema pallidum* infections are important causes of morbidity and mortality, and their elimination as global public health problems represents WHO goals for the achievement of the United Nations Agenda 2030 Sustainable Development Goals^(^ 8 ^-^ 10 ^)^.

In general, men are more susceptible to risk behavior and seek health services less when compared to women, especially those men belonging to more vulnerable populations with less education and purchasing power, including those living in rural areas^(^ 11 ^)^.

Male norms such as competitiveness, strength, power, and self-confidence can interfere with how a man whether commits or not to his own health^(^ 12 ^)^. Also, beyond gender issues, the operation hours of health facilities often coincide with the working hours of individuals, reducing their opportunities to participate in disease prevention and knowledge activities, and of their health situation and treatment^(^ 13 ^)^. In this context, screening and treating STIs in men is an important strategy for interrupting the chain of transmission of these infections, particularly among those who possibly have less access to health services, such as male field workers.

Brazil is one of the largest producers of sugarcane and alcohol in the world. Although mechanization of cane cutting is gradually replacing manual cutting, this technology has not yet fully reached some regions such as the Northeast and Midwest^(^ 14 ^)^. Manual sugarcane cutting is a strenuous job performed by men of low education and income. These individuals work under a productivity regimen and, in order to obtain better wage earnings, they submit themselves to an excessive workload. The seasonal nature of cane harvesting favors the migration of workers to harvesting regions^(^ 15 ^)^. Studies show several health problems related to sugarcane harvesting, among the most cited are respiratory problems, decreased kidney function, and repetitive strain injury^(^ 16 ^-^ 18 ^)^. However, there is no information on sexually transmitted infections in this population of rural, seasonal-migrant males.

In a previous publication, the prevalence of HIV, HBV, HCV and syphilis infections in the cane cutter population and analysis of factors associated with exposure to HBV were presented^(^ 19 ^)^. However, considering that these infections present common forms of transmission, the objective of the present study was to identify variables associated with these STIs (HIV, HBV, HCV, and syphilis), which can be used as indicators of vulnerability in sugarcane cutters operating in alcohol and sugar mills located in the Northeast and Midwest of Brazil.

## Method

This is an observational, cross-sectional study carried out in sugarcane mills in the states of Paraíba and Goiás, in Northeast and Midwest regions of Brazil, respectively. Data collection was conducted from February to September 2016.

During the study period, there were 38 and 9 sugar and alcohol mills in Goiás and Paraíba, respectively^(^ 20 ^)^. For the study, we considered the mills that performed predominantly manual harvesting of sugarcane and that were in the harvesting period. In Paraíba, data collection was carried out at the largest plant in the region, and in Goiás at four plants.

The target population of this study was rural workers, manual sugarcane cutters. All individuals aged 18 years or older, who performed manual cutting of sugarcane in alcohol and sugar mills in the State of Paraíba and Goiás were included. The minimum sample needed, considering a statistical power of 80% (β=20%), significance level of 5% (α<0.05), accuracy of 0.5%, 1.5 drawing effect, and prevalence for anti-HIV of 0.39%^(^ 21 ^)^ was 895 participants.

In general, cane cutters are distributed in teams of 30 to 50 men to perform the cane cutting. The recruitment of individuals was done in the cane field itself, during rest periods, sequentially and according to the arrival of the worker at the resting place. All eligible workers were invited to participate in the study and informed about its importance, objectives, risks, and benefits of participation, as well as the freedom to leave the study at any time. For individuals who wished to participate, the Informed Consent Form (ICF) was offered for reading and signing. In cases of illiterate ones, the ICF was read to the cane cutter and a witness, and the participant’s signature was fingerprinted.

Upon acceptance by the participant, the interview was conducted right in the sugarcane field, maintaining a minimum comfortable distance to respond with privacy. For this, we used a structured script adapted from the instrument used in the Research of Knowledge, Attitudes, and Practices in the Brazilian Population^(^ 22 ^)^, containing social demographic data and possible risk behaviors for the STIs investigated.

At the end of the interview, the participants were referred for rapid tests (RT) for HBV [HBsAg (Vikia HBsAg Biomerieux, France)], HCV [anti-HCV (Alere, Standard Diagnostics Inc, Republic of Korea)], syphilis [anti-*T. pallidum* (Alere Syphilis, Republic of Korea)] and HIV [anti-HIV-1 and 2 (screening - ABON HIV Tri-Line, Abon Biopharm, China; confirmatory - Bioeasy HIV, Republic of Korea)] as instructed by the manufacturers. We used rapid tests made available by the Brazilian Ministry of Health, through partnerships with the regional Health Secretariats. The interviewers and rapid test team were trained and prepared to perform these stages of the study.

For the analysis of the interview data and the rapid tests, the statistical STATA program version 13.0 (StataCorp., CollegeStation, TX) was adopted. The descriptive analysis was performed by frequency distribution, mean and standard deviation. We considered STI outcome the positive results in some of the tests performed. Prevalence was calculated with a 95% confidence interval (95% CI). Chi-squared and Fisher’s Exact tests were used to test differences in proportions. To estimate the odds ratio, the variables that presented a p-value < 0.20 were included in a logistic regression model^(^ 23 ^)^, using the *forward* procedure for selecting the variables. P-values< 0.05 associations were considered statistically significant The quality of the model adjustment was carried out through the *Hosmer-Lemershow test* (*p*=0.7007) and the ROC curve showed an area under the 0.75 curve.

This study was approved by the Research Ethics Committee of the Lauro Wanderley University Hospital of the Federal University of Paraíba according to protocol 1507737 and by the Research Ethics Committee of the Federal University of Goiás, according to protocols 1507737 and 042796/20, respectively. The ethical principles that guide research involving human beings, as described and established in Resolution nº466/2012 of the National Health Council, were respected at all stages of research^(^ 24 ^)^. Those individuals who tested positive for any infection were referred for diagnosis and/or treatment confirmation.

## Results

Of the total number of participants (n=937), 301 worked in sugar cane and alcohol mills in Paraíba and 636 in Goiás. The mean age was 35.4 years (SD=9.2 years) and most were under 40. We found that 47.4% declared schooling of fewer than five years. Regarding the place of birth, 85.7% were native of the Northeast Region. The average monthly income was R$ 1,801.00 (SD= R$ 438.00), and more than two thirds (71.2%) of the sugarcane cutters said they had religion (Table 1).

**Table 1:** Sociodemographic characteristics of sugarcane cutters in the states of Paraíba and Goiás (N = 937), Brazil, 2016

Variables	Paraíba (%)	Goiás (%)	Total (%)
	n=301	n=636	
Age* 35.4(9.2)^†^			
≤ 29	76 (25.2%)	190 (29.9%)	266 (28.4%)
30-39	121 (40.2%)	259 (40.7%)	380 (40.5%)
≥40	104 (34.6%)	187 (29.4%)	291 (31.1%)
Schooling* 5.2 (3.6)^†^			
≤ 4	183 (60.9%)	261 (41.0%)	444 (47.4%)
> 4	118 (39.1%)	375 (59.0%)	493 (52.6%)
**Marital status**			
Married/Stable union	274 (91.0%)	452 (71.0%)	726 (77.5%)
Single/Separated/Widow/Widower	27 (9.0%)	184 (29.0%)	211 (22.5%)
**Region of origin**			
Northeast	301 (100.0%)	502(79.0 %)	803 (85.7%)
Midwest	-	129 (20.3%)	129 (13.8%)
North	-	4 (0.6%)	4 (0.4%)
Southeast	-	1 (0.1%)	1 (0.1%)
**Income^‡^1801 (438)^†^**			
≤ 1500	139 (46.2%)	187 (29.4%)	326 (34.8%)
1500 - 2000	137 (45.5%)	275 (43.2%)	412 (44.0%)
> 2000	25 (8.3%)	174 (27.4%)	199 (21.2%)
**Religion**			
No	91 (30.2%)	179 (28.4%)	270 (28.8%)
Yes	210 (69.8%)	457 (71.6%)	667 (71.2%)

* years;

† mean (standard deviation);

‡ Reais (R$)/*per* month

By means of rapid tests, a prevalence in the set of these markers was estimated at 4.1% (95% CI: 3.0-5.5), that is, out of every 100 sugarcane cutters, four were positive to at least one of the STI.

The bivariate analysis showed a statistically significant association between positivity for some investigated STI and age, having a religion, reporting has had same-sex sexual intercourse, illicit drug use. These variables and those with a p-value< 0.20 (schooling, work region, number of sexual partnerships and prison experience) were included in a multiple logistic regression model, with the variables age (≥ 40 years; OR: 5;95% CI: 1,8-14), religion (OR: 0.4; 95% CI:0.2-0.8), Midwest Region (OR: 0.4;95% CI: 0.2-0.9), use of illicit drugs (OR: 2.9;95% CI: 1.3-6.3) and alcohol use (OR: 3.9;95% CI: 1.3-11.9) were significantly associated with the presence of STI.

**Table 2:** Bivariate and multiple analysis of factors associated with Sexually Transmitted Infections in sugarcane cutters in Paraíba and Goiás (N = 937), Brazil, 2016

	Bivariate Analysis		*p*	Multiple Analysis	*p*
	STI (n= 937)			(n=937)	
	Positive (n=38)	Negative (n=899)		OR* (95% CI)^†^	
	n (%)	n (%)			
**Age (years old)**					
≤ 29	5 (1.9%)	261 (98.1%)		1^‡^	
30 to 39	13 (3.4%)	367 (96.6%)	*0.248*	2.2 (0.8-6.4)	*0.138*
≥ 40	20 (6.9%)	271 (93.1%)	*0.008*	5(1.8-14)	*0.002*
**Schooling (years)**					
≤ 4	22 (4.95%)	422 (95.05%)		1	
> 4	16 (3.25%)	477 (96.75%)	*0.185*	1.1 (0.5-2.3)	*0.757*
**Marital status**					
Married/Stable union	27 (3.7%)	699 (96.3%)			
Single/Separated/Widow/Widower	11 (5.2%)	200 (94.8%)	*0.333*		
**Have a religion**					
No	17 (6.3%)	253 (93.7%)		1^‡^	
Yes	21 (3.2%)	646 (96.8%)	*0.027*	0.4 (0.2-0.8)	*0.015*
**Work region**					
Northeast	16 (5.3%)	285 (94.7%)		1^‡^	
Midwest	22 (3.5%)	614 (96.5%)	*0.179*	0.4 (0.2-0.9)	*0.028*
**Has shared housing during work**					
No	30 (4.6%)	630 (95.4%)			
Yes	8 (2.9%)	269 (97.1%)	*0.241*		
**Age of first sexual intercourse (years)**					
7 - 15 years	19 (4.1%)	444 (95.9%)			
>=16 years old	19 (4%)	455 (96%)	*0.941*		
**Same-sex sexual intercourse report**					
No	33 (3.7%)	852 (96.3%)		1	
Yes	5 (9.6%)	47 (90.4%)	*0.036*	1.9 (0.6-5.8)	*0.252*
**History of sexual abuse**					
No	37 (4%)	887 (96%)			
Yes	1 (7.7%)	12 (92.3%)	*0.503*		
**Number of sexual partnerships in the last 12 months**					
<= 1 partner	19 (3.4%)	545 (96.6%)		1	
>= 2 partners	19 (5.1%)	354 (94.9%)	*0.19*	1.7 (0.8-3.3)	*0.144*
**Has used condom in the last 12 months**					
Yes (always, sometimes)	22 (4.4%)	479 (95.6%)			
Never	16 (3.7%)	420 (96.3%)	*0.577*		
**Has used illicit drugs in life**					
No	28 (3.4%)	783 (96.6%)		1^‡^	
Yes	10 (7.9%)	116 (92.1%)	*0.018*	2.9 (1.3-6.3)	*0.008*
**Alcohol use (current)**					
No	4 (1.9%)	210 (98.1%)		1^‡^	
Yes	34 (4.7%)	689 (95.3%)	*0.065*	3.9 (1.3-11.9)	*0.017*
**Prison experience**					
No	32 (3.8%)	815 (96.2%)		1	
Yes	6 (6.7%)	84 (93.3%)	*0.187*	1.1 (0.4-3)	*0.826*
**Presence of tattoo and/or piercing**					
No	33 (4%)	798 (96%)			
Yes	5 (4.7%)	101 (95.3%)	*0.714*		

* OR = O*dds Ratio*;

† CI = 95% Confidence interval;

‡ Adjusted by: age, religion, work region, use of illicit drugs in life, use of alcohol

## Discussion

The rural population in Brazil lives, generally, in conditions of extreme poverty, isolation and in regions marked by socioeconomic inequalities and health vulnerabilities^(^ 25 ^)^. Especially, manual sugarcane cutters bear unhealthy working conditions, long working hours, high migratory flow and wages conditioned to productivity^(^ 26 ^)^. In this context, these workers have characteristics that expose them to various health hazards, including sexual infections. Few studies have been developed addressing the health status of sugarcane cutters^(^ 26 ^-^ 27 ^)^, and in the context of sexually transmitted infections, this is the first research that presents data on the prevalence of STIs and associated factors.

In line with other studies developed with manual sugarcane cutters, our population was marked by men, young and with few schooling years^(^ 27 ^-^ 28 ^)^. Young men predominance in this scenario is related to the type of work in sugarcane fields, which requires great effort and physical stamina^(^ 27 ^)^. On the other hand, the low schooling evidences the socioeconomic conditions of these workers^(^ 11 ^)^, and it has been related to less knowledge, less concern about health care, and consequently greater vulnerability in these individuals^(^ 29 ^)^. Gender and education represent explanatory variables for different risky sexual behaviors^(^ 29 ^-^ 30 ^)^.

In the present study, we observed that 4.1% (95% CI: 3.0-5.5) of cane cutters presented tests reagent on at least one of the STIs (HIV, syphilis, hepatitis B and C). Given the lack of studies showing the prevalence of a set of infections investigated in sugarcane cutters, data from this study were compared with those from other investigations conducted in rural populations, in which specific STIs were evaluated; and this highlights the need for programs to prevent and control these infections in rural workers. A national survey conducted in Rwanda, an African country, with 12,361 individuals living in rural areas, showed a prevalence of syphilis of 0.9%. (IC 95%: 0.7-1.1)^(^ 31 ^)^. In rural municipalities of China, it was observed that 3.0% (95% CI: 2.4-3.6) e 3.2% (95% CI: 2.6-3.8) of male clients of sex workers were positive for HIV and syphilis, respectively^(^ 32 ^)^.

In Brazil, in the rural region of São Paulo (Cássia dos Coqueiros), 1001 rural workers were investigated and 0.4% (95% CI:0.2-1.0) were positive for anti-HCV and 0.1% (95% CI:0.0-0.6) for HBsAg^(^ 33 ^)^. While a study conducted in Minas Gerais, with 757 residents of rural communities in Ouro Preto, detected rates for syphilis of 5.3% (95% CI:3.9-7.1), hepatitis B of 0.3% (95% CI:0.1-1.0), hepatitis C of 0.4% (95% CI:0.1-1.2) and no case of HIV was identified among the individuals investigated^(^ 34 ^)^.

As in other STI studies, a positivity gradient for STIs was observed with advancing age, with individuals over 40 years of age presenting five times more chance of positivity for any of the infections investigated when compared to those under 30 years of age. This result probably reflects lifetime exposures^(^ 35 ^-^ 36 ^)^.

One finding that drawn attention was the high frequency of alcohol and illicit drug consumption, and as observed in other studies^(^ 37 ^-^ 38 ^)^, these variables are positively associated with STIs. Almost all cane cutters reported consumption of alcohol and 13.4% of illicit drugs in their lives. The consumption of these drugs seems to be a reality among sugarcane cutters, often stimulated to withstand the excessive workload in the field^(^ 39 ^)^. Furthermore, the consumption of alcohol often promotes the individual’s disinhibition, in addition to reducing his or her memory, which contributes to reducing the perception of risk^(^ 40 ^)^. In fact, their daily consumption has been associated with inconsistent condom use and multiplicity of sexual partnerships^(^ 41 ^)^. Similarly, illicit drug use can encourage sexual risk behavior among users, making them vulnerable to STIs^(^ 42 ^)^. Such behaviors contribute to the acquisition and maintenance of the STIs’ transmission chain and constitute a serious public health problem.

Some studies have shown that practicing a religion reduces risk behaviors for STI by establishing behavioral norms for its followers^(^ 43 ^-^ 45 ^)^. In the present study, individuals who reported some religion presented lower positivity to STI, and this variable was negatively associated with these infections, reinforcing this hypothesis^(^ 46 ^-^ 47 ^)^.

Sugarcane cutters in activity in the Midwest Region presented a 60% lower chance of positivity to the group of investigated STIs. Most sugarcane cutters in Goiás (79%) are seasonal migrants from cities in the Northeast Region of the country, and have higher education levels than those from Paraíba (p< 0.001; data not presented). Thus, this condition may have contributed to the present finding, considering that education is a determining factor for social vulnerability, since it influences attitudes, risk recognition, adherence to disease prevention and treatment^(^ 48 ^-^ 49 ^)^.

Some limitations of this study must be considered. First, this is a cross-sectional study, i.e., it does not allow causal inference; however, it should be noted that the situational diagnosis of the population is the first step to propose interventions. Also, we considered for the presence of STIs the positivity for four sexual infections by means of rapid tests. It is known that several other pathogens are important in the sexual context, on the other hand, analyzing the predominance and silent behavior of HIV, *Treponema pallidum*, HBV, and HCV, it is justified the selection of these agents to compose the outcome variable of this study.

Finally, intimate questions (sexual and life habits) were used during the interview and many participants may have felt embarrassed to talk about these behaviors, and issued unreliable answers, however, at all times of the interview they were informed about the confidentiality of the information, for greater confidence in the answers received.

## Conclusion

The results show a prevalence of STIs in sugarcane cutters similar to rates in other rural communities around the world and Brazil. On the other hand, it points out risk behaviors for STI common to urban regions, such as alcohol and illicit drugs consumption. This scenario contributes to the risk of STI transmission and consequently the maintenance of the transmission chain of these infections. Thus, we recommend that sexual prevention integrate the actions to promote the health of sugarcane cutters in Brazil. Currently, sugar-alcohol companies are encouraged to ensure better working conditions, and among the various strategies are initiatives aimed at promoting workers’ health and safety. Rapid testing, together with a comprehensive approach to STIs, is an urgent and effective proposal to be implemented in this context of rural worker health prevention, as it provides a quick, easy and efficient way to prevent and diagnose STIs.
